# Combination therapy synergism prediction for virus treatment using machine learning models

**DOI:** 10.1371/journal.pone.0309733

**Published:** 2024-09-04

**Authors:** Shayan Majidifar, Arash Zabihian, Mohsen Hooshmand

**Affiliations:** 1 Department of Computer Science and Information Technology, Institute for Advanced Studies in Basic Sciences (IASBS), Zanjan, Iran; 2 Department of QA, Kimia Zist Parsian Pharmaceutical Company, Zanjan, Iran; University of St Andrews, UNITED KINGDOM OF GREAT BRITAIN AND NORTHERN IRELAND

## Abstract

Combining different drugs synergistically is an essential aspect of developing effective treatments. Although there is a plethora of research on computational prediction for new combination therapies, there is limited to no research on combination therapies in the treatment of viral diseases. This paper proposes AI-based models for predicting novel antiviral combinations to treat virus diseases synergistically. To do this, we assembled a comprehensive dataset comprising information on viral strains, drug compounds, and their known interactions. As far as we know, this is the first dataset and learning model on combination therapy for viruses. Our proposal includes using a random forest model, an SVM model, and a deep model to train viral combination therapy. The machine learning models showed the highest performance, and the predicted values were validated by a t-test, indicating the effectiveness of the proposed methods. One of the predicted combinations of acyclovir and ribavirin has been experimentally confirmed to have a synergistic antiviral effect against herpes simplex type-1 virus, as described in the literature.

## Introduction

Bioinformatics is an interdisciplinary domain among biology, mathematics, statistics, and computer science that tends to theoretically and practically explore the field of human health solutions [1]. In other words, it utilizes the notions and tools of computer science and engineering in the analysis or introduction of efficient solutions for working with biological, medical, and even pharmacological data and information. One of the aspects of bioinformatics is to assist the *drug discovery* industry [2]. This is because drug discovery is an expensive research area and always looks for methods that reduce the cost and time of proposing a new drug for a disease, especially in emergency situations [3]. The virus-based diseases like SARS-CoV-2 [4], Mpox [5], MERS-CoV [6, 7] confirm the necessity of introducing new treatments as fast as possible. However, the drugs need to be efficient with a low side effect [8]. To meet these goals, drug repurposing, a screening method, tries to locate new targets for the approved drugs [9]. First, it uses drugs that are approved, therefore have lower side effects, and can be trusted in treatments. Additionally, this approach narrows down the search space and consequently the cost and time for introducing the new drug. AI approaches, especially machine learning models, are commonly used in drug repurposing. The proposed drug repurposing methods cover a wide range of approaches from machine learning, e.g., logistic regression [10], random forest, support vector machine, neural networks [11–14], a spectrum of deep learning methods [15, 16] such as DTINet [17], NeoDTI [18], HIDTI [19], MolTrans [20], TransDTI [21]. Certain drug repurposing techniques have focused on predicting new associations between viruses and antivirals [22–27]. All previous methods have only considered single-drug treatments and have not explored the synergistic effects of combining multiple drugs.

However, each drug in addition to controlling and treating properties may have side effects and therefore increasing its usage dose causes high-risk issues in the patient [28]. Moreover, using a higher dose of a drug may cause *drug resistance* and nullify the treatment’s effectiveness [29]. Drug repurposing has another branch of drug-target association which uses more than one drug in the treatment of a target. It is called *combination therapy* [30] that tends to reduce the side effects of drugs, fix drug resistance, and more importantly increase the effect of the treatment, e.g., synergistic drug pairs [31]. Therefore, combination therapy aims to improve the treatment and drug efficacy [32].

The first method to check the efficacy of the combination of drug pairs is brute force search. No need to mention that this method is costly and uses a tremendous amount of time and resources. High-throughput screening is another approach to investigating combination therapy. Same as brute force it consumes time and resources tremendously. One approach to researching drugs for disease treatment is through computational methods that investigate the drug space and suggest drug pairs. Such machine learning methods have achieved significant prediction power in this research area [33].

Computational combination therapy in oncology is an enriched and hot topic nowadays [9, 34–38]. Preuer et al. used cancer cell line properties, i.e., gene expression, copy number, and gene mutation and drug information including, structural and molecular similarities and drug toxicity from Merck [34] and proposed a deep network to compute the synergistic score of combined drugs [36]. Zhang et al. used those entities from the NCI-ALMANAC [39] that have signaling pathways [37]. Zhang et al. [38] and Wang et al. [9] applied other deep models on new embeddings of cancer cell line properties. The former used autoencoder to drive new embeddings and the latter used kernel-based methods to extract meaningful features. Kuru et al. used two deep networks for embedding the generation of drugs and viruses from DrugCombo [40] and the new representations were fed to a third deep network for synergistic prediction of drugs for cancer treatment [41]. Julkunen et al. utilized the NCI-ALMANAC [39] dataset and mentioned that the previous works on drug combination in oncology had not considered protein properties and biological information of drugs. Then, they used factorization machines to decompose the information into latent spaces [42]. Meng et al. used a graph learning method to estimate the synergistic effect of combination therapy [43].

As mentioned earlier while combination therapy for oncology is a hot field, there are no general studies for virus treatments using synergistic therapy. Tan et al. proposed a multiplex screening method for HIV treatments [44]. This work does not use predictive learning models and targeted treatment of a single virus. Few studies proposed combination therapy solutions for SARS-CoV-2 [33, 45]. Although both works proposed combination therapy using deep models, they are limited to SARS-CoV-2 and have no general dataset for combination therapy.

This work proposes several machine learning methods for analyzing and evaluating virus-antivirals combination therapy. To accomplish this, we create a dataset containing the characteristics of both viruses and antivirals. Then, we devise and apply several machine learning methods to evaluate the effect of AI-based methods on the subject. The results are promising, and several new combined drugs for virus treatments are proposed. Based on our knowledge and the literature review, all research studies on virus treatment using combination therapy have been limited to experimental or single-virus treatment. Therefore, this is the first study on general virus combination therapy. The contribution of the paper is four-fold:

- First work on virus combination therapy.
- First complete dataset on virus combination therapy (CombTVir).
- Applying machine learning methods and evaluating the results.
- Applying t-test analysis for statistical analysis and prediction validation.
- Proposing new combined drugs for virus treatment. Some of these predictions have been confirmed in the literature.

The structure of the paper is as follows. Section *Dataset generation* describes the properties and aspects of the generated dataset. Section *Methods* introduces the proposed methods for combination therapy prediction. The results are reported in section *Results*. Section *Conclusion* concludes the paper.

## Dataset generation

This paper proposes a method for predicting effective antiviral combinations for treating viral diseases. The first step of this proposal is to find a suitable dataset that contains information on antivirals used for combination therapy. Unfortunately, there is currently no available dataset for viruses. Therefore, our paper’s first contribution is the creation of a virus combination therapy dataset, which we call it “CombTVir” dataset.

Myhre et al. gathered and reported a list of 541 drug combinations [46–48], of which 372 combinations belong to small molecule-small molecule (SM-SM) synergism, 103 combinations belong to biotech-biotech synergism, and the remaining 66 combinations belong to other types of combinations, e.g., SM-biotech. Notably, the combination list was sourced from PubMed or clinical trials. The selected combinations are derived from experiments *in vitro*, *in vivo*, or clinical trial phases. We chose those 372 SM-SM combined drugs for the dataset. Before describing the generation of the dataset, it is necessary to clarify the modifications made to the combination therapy list. The list contains HIV and HIV-1 (there were no reported HIV-2 in the list). After analyzing the main references of HIV and HIV-1, we treated HIV-1 as equivalent to HIV. Herpes simplex virus (HSV) has two subtypes—HSV-1 and HSV-2. These subtypes are highly similar genetically [49]. Since the dataset did not indicate the HSV subtype, we assumed HSV-1 and HSV are similar in this work. Some rows in the dataset are identical, such as the combination of acyclovir with foscarnet on HSV-1, which is repeated twice. The difference between the two rows is whether they were experimented on *in vitro* [50] or not reported [51].

We selected 372 SM-SM combinations from the dataset and removed all biotech-biotech and biotech-SM combinations, resulting in 44 viruses and 211 drugs being included in the chosen combinations. Table 1 briefly reports the statistics of the dataset. With these 372 SM-SM combinations, we gathered information about them from NCBI [52] and DrugBank [53]. NCBI is the National Center for Biotechnology Information which provides access to biomedical and genomic information. We gathered the Fasta version of viruses’ sequences from NCBI. DrugBank is a freely accessible database that contains information on drugs and their targets; therefore, we collected the SMILES [54] of drugs from it. Thus, we have information on drugs and viruses.

**Table 1:** Combination therapy (CombTVir) dataset.

Entity	Statistics
No of Viruses (V)	44
No. of antivirals (A)	211
No of approved interactions (AAV)	372
Sparsity	0.019%

In the next step, we prepared the feature vector of each antiviral and each virus by creating similarity matrices for antivirals and viruses. To compute the drugs’ similarity matrix, as Bajusz et al. [55] suggested, we converted the SMILES of antivirals to fingerprints and then applied the following Tanimoto score [56] on each fingerprint pair.

$$\begin{array}{c} Tanimoto\left(A,B\right)=\frac{\left|A\cap B\right|}{\left|A|+|B|-|A\cap B\right|} \end{array}$$ (1)

Then, the feature vector of each antiviral is its Tanimoto scores with whole antivirals. Consequently, their generated similarity matrix acts as the feature set of antivirals.

As mentioned earlier, we gathered the Fasta sequences of viruses from NCBI by choosing the complete genome version of the virus or its first row from the RefSeq section. Thus, we gathered the sequences of 44 viruses. Then, to prepare the viruses’ feature vectors, we calculated their similarity matrix using sequence alignment [57]. We implemented the Smith-Waterman algorithm [58], a pairwise sequence alignment method, on every pair of sequences using the NUC44 score matrix. This algorithm takes two strings and aligns them to maximize the alignment score. It works as follows.

$$\begin{array}{c} S_{i,j}=\text{max}\{\begin{array}{cc} S_{i-1,j-1}+s\left(a_{i},b_{j}\right) & ↖\\ S_{i,j-1}+s\left(a_{i},-\right) & \leftarrow \\ S_{i-1,j}+s\left(-,b_{j}\right) & ↑ \end{array} \end{array}$$ (2)

Where, **a** and **b** are two strings with lengths of *m* and *n*, respectively. The first row of the equation states that when the *i*-th character of **a** and *j*-th of **b** match, the total score increases. When there is no match, the maximum value based on insertion or deletion is computed using the second or third row. The algorithm returns the value of *S*(*m*, *n*) as its alignment score [59]. These scores are considered as the entries of each virus feature vector. In other words, we compute the sequence alignment scores for each virus and generate the similarity matrix based on them. Then, each row of the similarity matrix is considered as the feature vector of its corresponding virus. Having these two similarity matrices and the list of available combination therapies, we have prepared the CombTVir dataset for further analysis in the next sections.

## Methods

**Fig 1:**
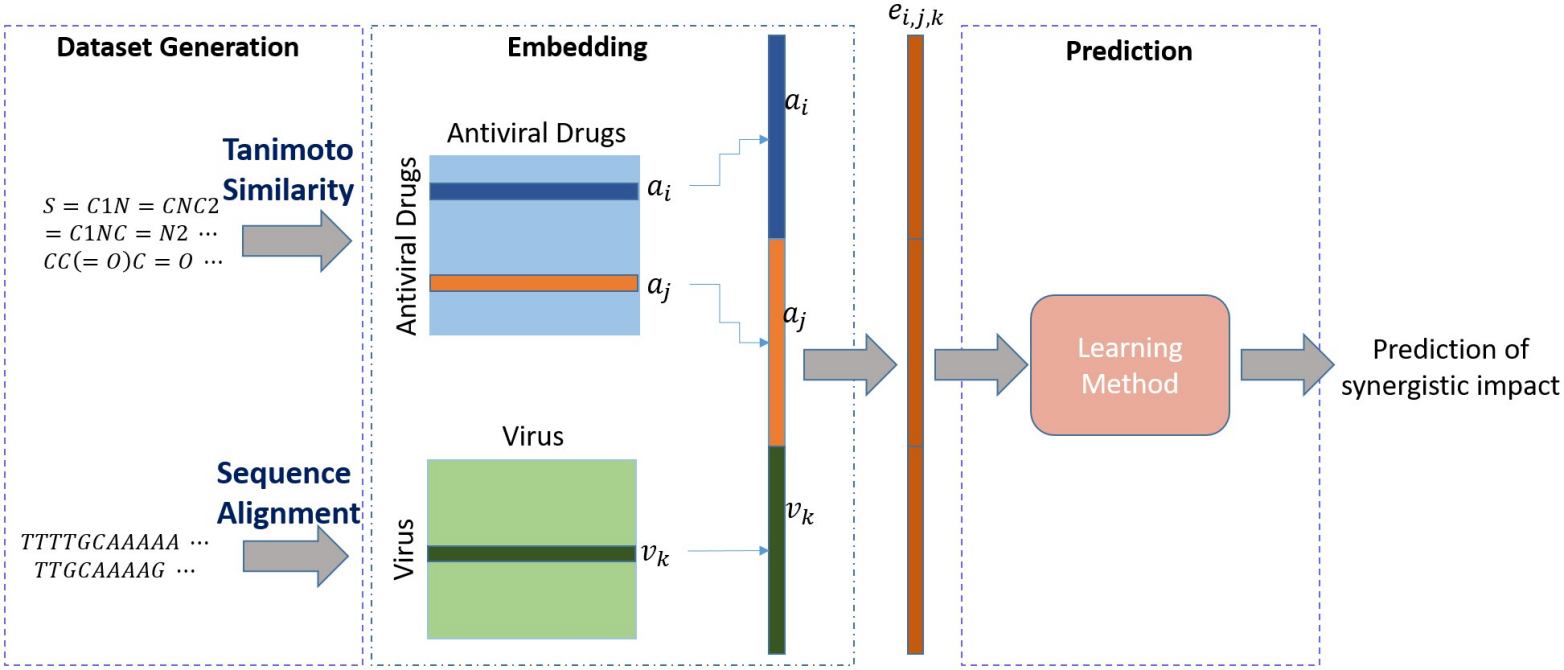
Drug combination learning framework. The framework prepares embeddings for each drug and each target based on their similarity information. Then, the corresponding embeddings of each drug-drug-target combination are concatenated, which is the input of the prediction step. The final step uses one of the proposed learning methods, i.e., SVM, RF, and DRaW to predict the interaction of each pair.

As mentioned in the previous section, the dataset consists of the antiviral feature set *A*, the virus feature set *V*, and the antiviral-antiviral-virus associations *Y*. We consider the latter as labels. Having this, we aim at predicting the synergistic effect of combining two antivirals *i* and *j*, where **a**_*i*_, **a**_*j*_ ∈ *A* on the given virus *k*, **v**_*k*_ ∈ *V* using support vector machine, random forest, and a deep model which we call DRaW. Fig 1 shows the general framework of the proposed methods. The antiviral set *A* contains *m* antivirals and the virus set *V* contains *n* viruses. To use the machine learning methods, each identity of the problem, i.e., antiviral and virus, needs a corresponding feature vector. As mentioned in the previous section, the feature set is extracted from the similarity vectors of antivirals and viruses. The final feature vector of each combination is the fruit of concatenation of *i*-th and *j*-th antivirals(**a**_*i*_ and **a**_*j*_) and *k*-th virus, or **e**_*i*,*j*,*k*_ = **a**_*i*_ ‖ **a**_*j*_ ‖ **v**_*k*_. Therefore, the vector **e**_*i*,*j*,*k*_ represents the feature vector of *i*-th and *j*-th antivirals, and *k*-th virus with the aim of predicting the label *y*_*i*,*j*,*k*_ ∈ *Y* using the mentioned feature vector to minimize the general loss function as follows:

$$\begin{array}{c} \text{min}\;L=\text{min}(\sum_{i=1}^{m}\sum_{j=1,j\neq i}^{m}\sum_{k=1}^{n}dist(y_{i,j,k},{\hat{y}}_{i,j,k})). \end{array}$$

Where, *y*_*i*,*j*,*k*_ shows the label of the synergistic effect of drugs *i*, *j* on virus *k*. ${\hat{y}}_{i,j,k}$ shows its predicted version and is computed using an effective learning method. The function *dist*(⋅, ⋅) is the distance function for the evaluation of the learning methods. As discussed earlier, combination therapy uses several learning methods from the literature. We use SVM [11], random forest [12] for their high performance in different domains of learning [13, 14], and a convolutional deep learning model due to their efficiency, performance, and reliability [60].

### Support vector machine

The support vector machine (SVM), introduced by Vapnik, finds the margin with the maximum length between two classes of data. However, since the two classes are not always linearly separable, various techniques such as the use of kernels and misclassification allowance are employed for optimal classification. Therefore, its loss function is as follows.

$$\begin{array}{c} \text{min}\;L=\underset{G,\text{b}}{\text{min}}\frac{1}{2}{\left\|G\right\|}^{2}+C\sum_{i,j,k,i\neq j}\varepsilon_{i,j,k}, \end{array}$$ (3)

$$\begin{array}{c} y_{i,j,k}(G^{t}{\text{e}}_{i,j,k}+b)\geq 1-\varepsilon_{i,j,k},\forall i,j\in \left\{1,\cdots ,m\right\},k\in \left\{1,\cdots ,n\right\} \end{array}$$ (4)

Where, *G* is support vectors, *C* is error regulation parameter, *ε* is the allowed error [61].

### Random forest

Random forest (RF) is an ensemble machine learning method that utilizes several decision trees and each tree randomly chooses several features from the feature sets. Following the learning phase of the trees, the class with the majority vote is chosen as the predicted label. Using several trees and a random selection of features for each tree leads to neutralizing the overfitting effect of decision trees. More importantly, the ensemble of trees yields a reliable prediction result of random forest. This principle makes a random forest a high-performance ML method for classification. In this work, the decision trees use *Gini* and *logloss* functions for score computation in each level of trees [62].

### DRaW–a deep learning method

**Fig 2:**
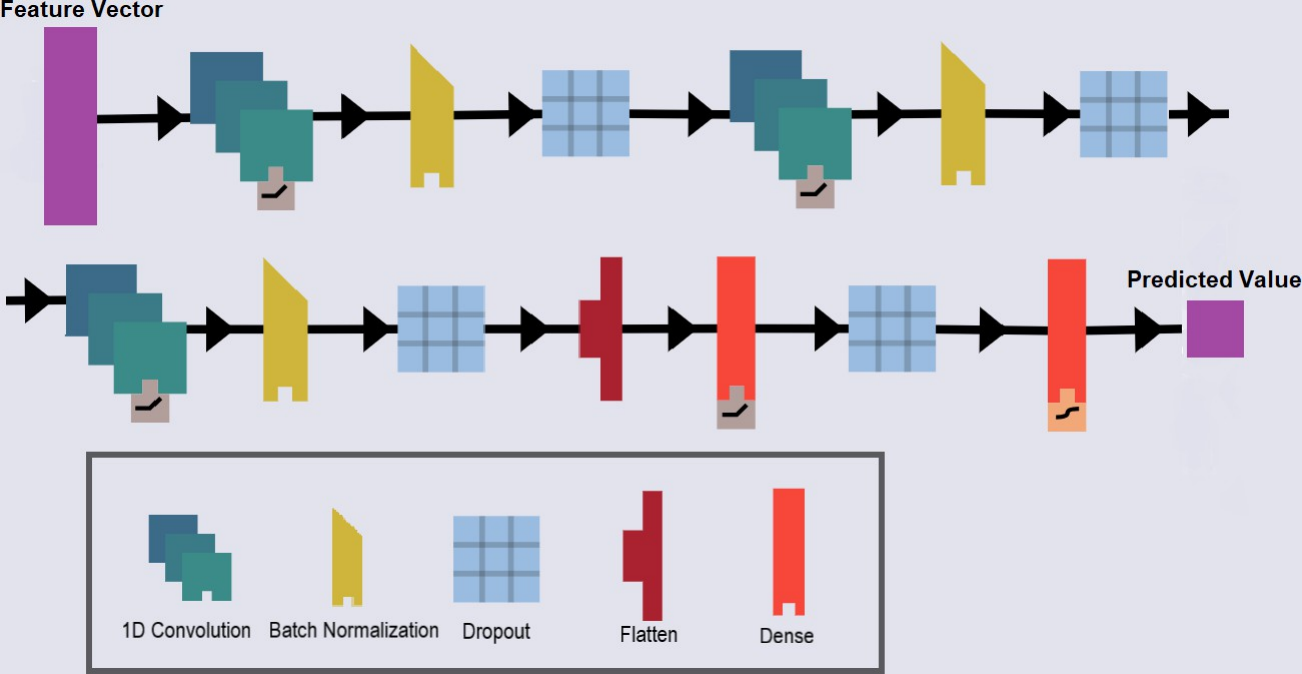
The DRaW deep model consists of three convolution layers, each containing a convolution, a batch normalization, and a dropout module. The activation function used in the inner layers is RelU. Finally, the last layer is the classification module, which uses a sigmoid activation function for classification.

Fig 2 shows the architecture of the proposed deep model, DRaW. It consists of three CNN layers and each CNN layer is a combination of 1D convolution, batch normalization, and dropout layers. After the CNN layers, there are two dense layers with a dropout layer in between. All the internal activation functions are *ReLU* and the last layer activation function is a *sigmoid* function. DRaW accepts the **e**_*i*,*j*,*k*_ as input feature vectors and computes their corresponding ${\hat{y}}_{i,j,k}$.

$$\begin{array}{c} {\hat{y}}_{i,j,k}=DRaW\left({\text{e}}_{i,j,k}\right) \end{array}$$

Its loss function is binary cross-entropy on all members of the dataset.

$$\begin{array}{c} minL=\text{min}(-\sum_{i=1}^{m}\sum_{j=1,j\neq i}^{m}\sum_{k=1}^{n}[y_{i,j,k}\;\text{log}\left({\hat{y}}_{i,j,k}\right)+(1-y_{i,j,k})\;\text{log}\;(1-{\hat{y}}_{i,j,k})]). \end{array}$$ (5)

Algorithm 1 presents the DRaW algorithm. The inputs are the antiviral-antiviral-virus label set *Y*, the antiviral feature matrix *A*, and the virus feature matrix *V*. Additionally, the algorithm needs the initiation of three more parameters of *ratio*, *folds*, and *epochs*. The *ratio* determines the positive-to-negative(P-to-N) sampling ratio. The *folds* parameter sets the number of folds for k-fold stratification, and finally, *epochs* identifies the number of iterations. The output is the predicted associations $\hat{Y}$. The algorithm in line 1 chooses a sample set of labels based on the *ratio* parameter. It chooses the whole positive samples, and a random number of negative samples using the ratio. For instance, when the sampling ratio is set to 1: 10, the algorithm randomly selects ten negative samples for every positive sample. Then, the algorithm employs a stratified version of the k-fold cross-validation procedure, and line 2 demonstrates the data folding process based on the *folds* parameter. The main section of the algorithm starts from line 3 and goes on. For each fold, the data is split into training and test sets based on the corresponding fold in line 4. The model is trained based on the features and labels of the training set in line 6, and this training is done in several epochs. The DRaW model in Fig 1 serves as the basis for this training. Line 7 predicts the test labels. Line 8 of the algorithm computes the loss function based on the binary cross entropy loss function introduced in Eq 5. After, ending the epochs, the algorithm predicts the test set labels. In the end, the algorithm calculates the performance based on the evaluation metrics presented in Section.

**Algorithm 1** Proposed Deep Model (*DRaW*)

**Input**: *A*, *V*, *Y*, *ratio*, *folds*, *epochs*

**Output**: $\hat{Y}$

1: data split(Y, ratio)

2: k-Fold ← stratified-k-Fold(folds)

3: **for** each fold in k-Fold **do**

4:  divide data into train and test

5:  **For** each epoch in epochs **do**

6:   *Model* = *Training*(*A*_*tr*_, *V*_*tr*_, *Y*_*tr*_)

7:   $\hat{Y}=Compute\left(A_{ts},V_{ts},Y_{ts}\right)$

8:   Loss computation using Eq 5

9:  **end for**

10: **end for**

11: Performance evaluation

### Complexity analysis

Assuming a dataset with *m* antivirals and *n* viruses, the complexity analysis is divided into two parts: dataset preparation and generation of feature vectors for antivirals and viruses. As mentioned earlier, we used the Tanimoto score and *sequence alignment* score to create the similarity matrices. The Tanimoto score is used to measure the similarity between two sets, while the sequence alignment score is used to measure the similarity between two sequences. The Tanimoto score complexity is *cn*^2^, where c is a small constant. This means that the runtime is fast, as a result, the entire procedure can be completed in just a fraction of a second. Performing pairwise sequence alignment for all viruses is a task that takes a considerable amount of time. The complexity of this algorithm is *Cn*^2^, where *C* is a huge constant, therefore, it is a time-consuming computation. SVM training time complexity is in the range *O*(*m*^2^*n*^2^) and *O*(*m*^3^*n*^3^) depending on the *C* hyperparameter and its runtime is *O*(|*G*|*mn*), where |*G*| is the number of support vectors [63]. The time complexity of random forest uses N trees each with at most V sampled features [64]. Therefore, its training time complexity is *O*(*NVmn*(log *m* + log *n*). Its runtime is *O*(*Nd*), where *d* is the depth of the tree. The DRaW runs for *E* epochs of each *T* long. Therefore, its time complexity is *O*(*mnET*) asymptotically.

## Results

This section provides the results of the proposed methods of virus-antiviral combination therapy. We performed 10-fold stratified cross-validation on a system with Ubuntu 22.04 LTS operating system. The system runs on an Intel Xeon Processor E5 v4 family with 4 CPU threads, 16 GB of RAM, and 20 GB of storage capacity.

The performance evaluation metrics are as follows.

$$\begin{array}{c} \text{Accuracy}=\frac{TP+TN}{TP+TN+FP+FN}, \end{array}$$ (6)

$$\begin{array}{c} MCC=\frac{TP\times TN-FP\times FN}{\sqrt{\left(TP+FP)(TP+FN)(TN+FP)(TN+FN\right)}}, \end{array}$$ (7)

$$\begin{array}{c} \text{AUC-ROC}=\sum_{i=1}^{n}TPR_{i}\Delta FPR_{i}, \end{array}$$ (8)

$$\begin{array}{c} \text{AUPR}=\sum_{i=1}^{n}{\text{precision}}_{i}\;\Delta \;{\text{recall}}_{i}. \end{array}$$ (9)

Moreover, we conducted a t-test on the predicted results [65] to evaluate the domain applicability and statistical analysis. We assume the null hypothesis H0 results from a lack of correlation between the original and predicted labels. The alternative hypothesis states the existence of a correlation between the two sets. Large values of p-value confirm the H0 and small values reject it.

We conducted the simulation for several P-to-N sampling ratios, i.e., 1:3, 1:5, 1:10, 1:100, and 1:500. For the lower sampling ratios—1:10 and lower ones– the performance of all methods is almost equal and close to perfect. Therefore, we report the results for the sampling ratios of 1:100 and 1:500. In our study, we performed a grid search on various configurations of SVM and random forest to identify the optimal performance of these ML methods. For SVM, we analyzed three different kernels (Linear, Poly, and RBF) and evaluated three different values of C for each kernel. The results of this analysis are provided in S4-S6 Tables in S1 File for sampling ratios 1:10, 1:100, and 1:500, respectively. As the results show, the SVM with specifications “poly” kernel and C = 10 has the best performance. Therefore, we use this model of SVM for comparison with other learning models. Additionally, we evaluated the random forest model using two criteria, *Gini* and logloss. We also tested two different values for the maximum number of features for each criterion. The results of these analyses are presented in S9-S11 Tables in S1 File for sampling ratios 1:10, 1:100, and 1:500, respectively, in the S1 File. The results confirm that the random forest with the *logloss* criteria and a maximum number of features for *log(n)* has been chosen for comparison with other learning models. These configurations for RF and SVM were then used for general comparison purposes.

Table 2 shows the metric scores for DRaW, SVM, and random forest for the P-to-N sampling ratio of *1:100*. While all methods have the same accuracy, The SVM has the highest AUC-ROC and the random forest has the highest AUPR.

**Table 2:** Results for positive-to-negative sampling ratio of 1:100.

Method	DRaW	SVM	RF
Acc	**0.99** ± **0.0**	**0.99** ± **0.0**	**0.99** ± **0.0**
MCC	0.87 ± 0.02	0.85 ± 0.03	**0.92** ± **0.02**
AUC-ROC	0.98 ± 0.01	**0.99** ± **0.0**	0.97 ± 0.02
AUPR	0.86 ± 0.04	0.92 ± 0.03	**0.93** ± **0.04**

Table 3 reports the results for the P-to-N sampling ratio of *1:500*. The same pattern similar to Table 2 happens for this ratio as well.

**Table 3:** Results for P-to-N sampling ratio of 1:500.

Method	DRaW	SVM	RF
Acc	**0.99** ± **0.00**	**0.99** ± **0.0**	**0.99** ± **0.0**
MCC	0.77 ± 0.04	0.72. ± 0.06	**0.78** ± **0.05**
AUC-ROC	0.92 ± 0.03	**0.97** ± **0.01**	0.92 ± 0.02
AUPR	0.69 ± 0.06	0.75 ± 0.07	**0.78** ± **0.05**

**Fig 3:**
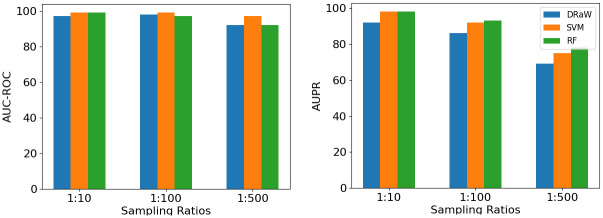
AUC-ROC and AUPR values of different methods. The x-axis displays P-to-N sampling ratios while the y-axis represents AUC-ROC and AUPR values for the left and right plots. (A) The AUC-ROC value of SVM remains stable and almost constant even when the sampling ratio increases. (B) In contrast, the right plot shows a decrease in the AUPR value of all methods. For higher sampling ratios, the AUPR value and overall performance of the random forest remain higher than other methods.

Visual comparison of AUC-ROC and AUPR for different methods is presented in Fig 3. Results are reported for three P-to-N sampling ratios: 1:10, 1:100, and 1:500. In this study, we compared the changes in AUC-ROC when varying the sampling ratio. Fig 3A shows that the AUC-ROC scores for 1:10 and 1:100 remain almost unchanged, regardless of whether DRaW, SVM, or RF are used. The ML methods outperform the deep model regarding the mentioned evaluation metric. Among the ML methods, the SVM has the highest AUC-ROC. However, all methods show a decrease in performance when increasing the sampling ratio to 1:500. In the figure labeled as Fig 3B, we can see the AUPR (Area Under the Precision-Recall Curve) of different methods for different P-to-N sampling ratios. As the P-to-N sampling ratio increases, there is a decrease in the AUPR scores of all methods. It is observed that DRaW has a lower score compared to ML (Machine Learning) methods for the whole sampling ratios. Random forest is the top performer based on AUPR for all sampling ratios.

The validation of the proposed model is crucial for generalization and checking the suggested combinations. Therefore, we conducted a t-test statistical analysis to validate the prediction models. Table 4 shows the t-test results of the predicted values. It reports the significance for sampling ratios of 1:10, 1:100, and 1:500 for all methods, i.e., DRaW, SVM, and random forest. We set the threshold to 0.05. All predicted values have p-values below the threshold and reject the null hypothesis.

**Table 4:** t-test validation.

Ratio	SVM	RF	DRaW
1:10	5.71E-07	1.91E-11	3.5E-156
1:100	1.0E-100	4.59E-41	1.09E-81
1:500	1.0E-100	6.57E-94	9E-146

**Fig 4:**
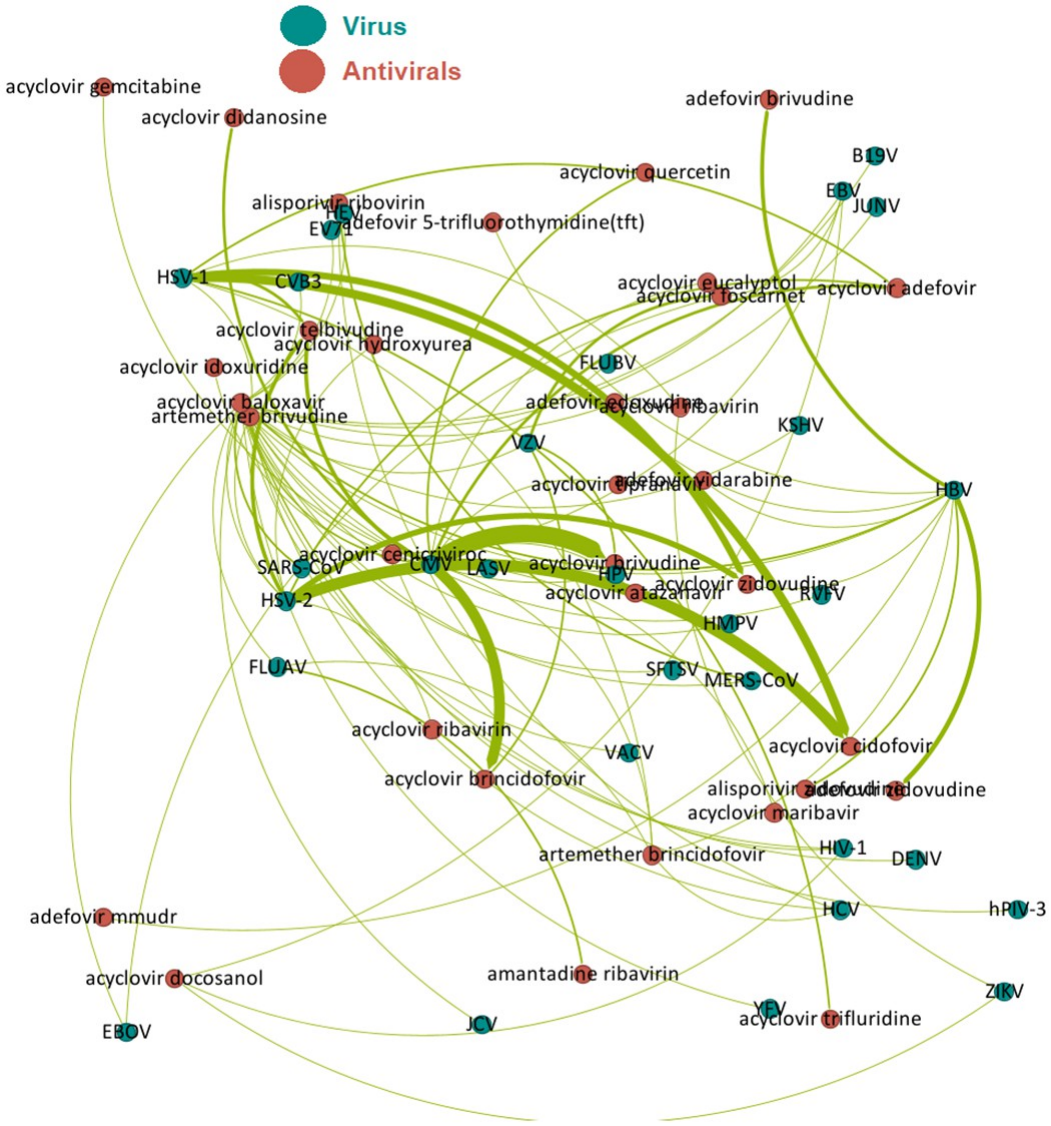
Predicted synergistically combined antivirals on viruses.

The results demonstrate that the proposed methods effectively predict synergistic combinations of antiviral drugs. Therefore, we present the predicted combinations of antiviral drugs that are effective against previously unknown viruses. Fig 4 illustrates a schematic graph of the proposed antiviral drug combinations.

In order to validate the results, we conducted a literature search to identify antiviral drug combinations that have individually demonstrated effectiveness in treating specific viruses. For instance, while acyclovir and brincidofovir have shown treatment efficacy for CMV, our model suggests that combining the two could produce a synergistic effect. However, this proposed effect will need to be confirmed by future experimental studies.

Another prediction of the proposed model is the synergistic effect of acyclovir and cidofovir on HSV-1. Both of these medications are individually effective treatments for the mentioned virus. The literature also indicates that the combination of acyclovir and zidovudine has an additive effect on HSV-1, which the model also predicted to have a synergistic effect. Acyclovir and foscarnet have an additive effect on VZV, where our proposed machine learning models predict their synergistic treatment [66]. The additive combination of acyclovir and maribavir on CMV is predicted to have a synergistic treatment [67]. Additionally, it is predicted that acyclovir in combination with trifluridine and adefovir has a synergistic effect on treatments for HSV-1, and in combination with brincidofovir and brivudine has a synergistic effect on VZV. The model predicts that alisporivir and ribavirin have a synergistic effect on HCV and their additive effect has been confirmed experimentally. Clinical trials are necessary for the validation of these new combinations. Table 5 reports those predictions which at least have an additive treatment for viruses. Additionally, S13 and S14 Tables in S1 File report the complete list of unknown synergistic combination therapies against viruses predicted with proposed methods. The frequency shows the number of predictions in test sets.

**Table 5:** Predicted synergistic combinations of antivirals. Each citation reports the efficacy of its corresponding antiviral against the virus. The complete list of predicted combinations is available in the S1 File. Note that the synergistic effect of Acyclovir and Ribavirin against HSV-1 has been confirmed.

Antiviral1	Antiviral2	Virus
Acyclovir [69]	Ribavirin [70]	HSV-1 [68]
Acyclovir [71]	Cidofovir [72]	HSV-2
Acyclovir [66]	Brincidofovir [73]	CMV
Acyclovir [69]	Cidofovir [72]	HSV-1
Acyclovir [69]	Zidovudine [74]	HSV-1
Acyclovir [66]	Foscarnet [75]	VZV
Acyclovir [69]	Trifluridine [76]	HSV-1
Acyclovir [66]	Brincidofovir [77]	VZV
Acyclovir [66]	Brivudine [78]	VZV
Acyclovir [69]	Adefovir [79]	HSV-1
Alisporivir [80]	Ribavirin [81]	HCV
Acyclovir [66]	Maribavir [67]	CMV
Acyclovir [82]	Foscarnet [75]	EBV

More importantly, one of the predicted combinations, i.e., the synergistic effect of acyclovir and ribavirin against the herpes simplex type-1 virus (HSV-1) has been confirmed experimentally [68].

## Conclusion

This paper proposes machine learning models to predict the synergistic effects of antiviral combinations on viruses. While synergistic combination therapy has a rich history of research, to the best knowledge of the authors there is no research on computational combination therapy for viruses. Therefore, in this paper, we have proposed a first dataset for the virus synergistic combination therapy. Moreover, we conducted several learning methods including random forest, SVM, and a deep model for efficient prediction of the synergistic effect of combined antivirals on the virus. The results confirm the high performance of all proposed methods. The results show the high performance of the random forest model. Increasing the sampling ratios notably resulted in the random forest having the best performance. In the future, using attention-based learning methods to model synergistic viruses can improve results. Additionally, the feature vectors are similarity vectors of antivirals and viruses. The similarity vectors are based on linear operators like cosine similarity. Therefore, using the similarity vector can impact and decrease the effect of learning models. Therefore, the direct feeding of SMILES of antivirals can improve the performance of learning models. Combining the self-attention methods with different ways of preparing the input features is another area for further research.

This paper confirms the results by applying a t-test to the predicted results and rejecting the null hypothesis. Experimental analysis is required to validate proposed drug combinations and determine if their effects are additive or synergistic. One combination (not in the dataset), acyclovir and ribavirin, was successfully predicted and approved in the literature against HSV-1. It is worth mentioning that acyclovir shows up in most of the predictions. This is due to its frequent presence in most approved synergistic combination actions.

## Supporting information

S1 File The supplementary material contains comprehensive information on machine learning hyperparameter optimization, comprehensive results, and predicted combinations. (PDF)
