# Supplementary material for: Combination therapy synergism prediction for virus treatment using machine learning models
Source: PLoS One. 2024 Sep 4;19(9):e0309733. doi: 10.1371/journal.pone.0309733 (PMC11373828; doi:10.1371/journal.pone.0309733)
Supplement: S1 File — (PDF) [file pone.0309733.s001.pdf]

Combination Therapy Synergism Prediction for Virus Treatment Using Machine and Deep Learning Models: *supplementary material*

Shayan Majidifar[1], Arash Zabihian[2], Mohsen Hooshmand[1*]

1 Department of Computer Science and Information Technology, Institute for Advanced Studies in Basic Sciences (IASBS), Zanjan, Iran
2 Department of QA, Kimia Zist Parsian Pharmaceutical Company, Zanjan, Iran
* mohsen.hooshmand@iasbs.ac.ir

Abstract

The document contains supplementary information related to the paper of the same title. Section 1 details an exhaustive search through a subset of the hyperparameter space of the proposed ML algorithms. Furthermore, Section 2 provides excessive information on the evaluation of ML-proposed methods on a proposed dataset. In the next section 3, complete results of the deep learning model for 5 positive to negative ratios are shown. Finally, Section 4 reports combinations that the proposed methods predicted.

1 Machine learning hyperparameter optimization

Table 1 reports the different values of the parameters tested for each one of the machine learning models during the process of hyperparameter optimization.

Table 1. ML parameters examined throughout the grid search

ML Algorithm	Parameters	Parameters' Values
Random Forest	Criterion	{gini, entropy, log loss}
	Max No. Features	{$\sqrt{n}$, $\log_2 n$ }
SVM	Kernel	{linear, poly, RBF}
	C	{0.1, 1, 10}

2 Machine learning results on the *CombTVir* dataset

This section evaluates SVM and RF performance on positive:negative samples— i.e., 1:3, 1:5. 1:10, 1:100, 1:500— of the *CombTVir* dataset in Tables 2 to 11.

Table 2. SVM method performance evaluation on 1:3 positive to negative sampling ratio of the CombTVir dataset.

Kernel	C	Acc	MCC	AUC-ROC	AUPR
Linear	0.1	0.98 ± 0.01	0.94 ± 0.03	**0.99 ± 0.0**	**0.99 ± 0.0**
	1	**0.99 ± 0.0**	0.97 ± 0.01	**0.99 ± 0.0**	**0.99 ± 0.0**
	10	0.98 ± 0.0	**0.99 ± 0.01**	**0.99 ± 0.0**	**0.99 ± 0.0**
Poly	0.1	**0.99 ± 0.0**	0.98 ± 0.02	**0.99 ± 0.0**	**0.99 ± 0.0**
	1	0.98 ± 0.0	0.97 ± 0.02	**0.99 ± 0.0**	**0.99 ± 0.0**
	10	0.98 ± 0.0	0.96 ± 0.0	**0.99 ± 0.0**	**0.99 ± 0.0**
RBF	0.1	0.97 ± 0.01	0.93 ± 0.03	**0.99 ± 0.0**	0.98 ± 0.01
	1	0.98 ± 0.01	0.96 ± 0.02	**0.99 ± 0.0**	**0.99 ± 0.0**
	10	0.98 ± 0.01	0.97 ± 0.02	**0.99 ± 0.0**	**0.99 ± 0.0**

Table 3. SVM method performance evaluation on 1:5 positive to negative sampling ratio of the CombTVir dataset.

Kernel	C	Acc	MCC	AUC-ROC	AUPR
Linear	0.1	0.98 ± 0.01	0.94 ± 0.03	**0.99 ± 0.0**	**0.99 ± 0.0**
	1	0.98 ± 0.01	0.94 ± 0.03	**0.99 ± 0.0**	**0.99 ± 0.0**
	10	**0.99 ± 0.0**	**0.97 ± 0.02**	**0.99 ± 0.0**	**0.99 ± 0.0**
Poly	0.1	**0.99 ± 0.0**	0.96 ± 0.03	**0.99 ± 0.0**	**0.99 ± 0.0**
	1	**0.99 ± 0.0**	0.96 ± 0.02	**0.99 ± 0.0**	**0.99 ± 0.0**
	10	0.98 ± 0.0	0.96 ± 0.03	**0.99 ± 0.0**	**0.99 ± 0.0**
RBF	0.1	0.98 ± 0.01	0.93 ± 0.04	**0.99 ± 0.0**	0.98 ± 0.01
	1	0.98 ± 0.01	0.95 ± 0.04	**0.99 ± 0.0**	**0.99 ± 0.0**
	10	**0.99 ± 0.0**	**0.97 ± 0.02**	**0.99 ± 0.0**	**0.99 ± 0.0**

Table 4. Performance evaluation of the different configurations of the SVM model on a 1:10 sample of the CombTVir dataset

Kernel	C	Acc	MCC	AUC-ROC	AUPR
Linear	0.1	0.98 ± 0.0	0.89 ± 0.03	**0.99 ± 0.0**	0.98 ± 0.01
	1	0.98 ± 0.0	0.93 ± 0.03	**0.99 ± 0.0**	**0.99 ± 0.0**
	10	**0.99 ± 0.0**	0.94 ± 0.03	**0.99 ± 0.0**	**0.99 ± 0.0**
Poly	0.1	0.98 ± 0.0	0.93 ± 0.03	**0.99 ± 0.0**	**0.99 ± 0.0**
	1	**0.99 ± 0.0**	0.95 ± 0.02	**0.99 ± 0.0**	**0.99 ± 0.01**
	10	**0.99 ± 0.0**	**0.96 ± 0.02**	**0.99 ± 0.0**	0.98 ± 0.01
RBF	0.1	0.98 ± 0.0	0.93 ± 0.03	**0.99 ± 0.0**	0.97 ± 0.01
	1	**0.99 ± 0.0**	0.91 ± 0.02	**0.99 ± 0.0**	**0.99 ± 0.0**
	10	0.99 ± 0.0	0.94 ± 0.02	**0.99 ± 0.0**	**0.99 ± 0.0**

Table 5. Performance evaluation of the different configurations of the SVM model on a 1:100 sample of the CombTVir dataset

Kernel	C	Acc	MCC	AUC-ROC	AUPR
Linear	0.1	0.96 ± 0.0	0.41 ± 0.01	0.98 ± 0.0	0.79 ± 0.04
	1	0.96 ± 0.0	0.45 ± 0.02	0.98 ± 0.0	0.8 ± 0.05
	10	0.97 ± 0.0	0.51 ± 0.03	0.98 ± 0.0	0.82 ± 0.05
Poly	0.1	0.98 ± 0.0	0.67 ± 0.02	**0.99 ± 0.0**	0.89 ± 0.03
	1	**0.99 ± 0.0**	0.81 ± 0.03	**0.99 ± 0.0**	0.91 ± 0.04
	10	**0.99 ± 0.0**	**0.85 ± 0.03**	**0.99 ± 0.0**	**0.92 ± 0.03**
RBF	0.1	0.97 ± 0.0	0.49 ± 0.02	**0.99 ± 0.0**	0.86 ± 0.02
	1	**0.99 ± 0.0**	0.68 ± 0.02	**0.99 ± 0.0**	0.88 ± 0.03
	10	**0.99 ± 0.0**	0.8 ± 0.04	**0.99 ± 0.0**	0.91 ± 0.03

Table 6. Performance evaluation of the different configurations of the SVM model on a 1:500 sample of the CombTVir dataset

Kernel	C	Acc	MCC	AUC-ROC	AUPR
Linear	0.1	0.93 ± 0.0	0.14 ± 0.00	0.95 ± 0.01	0.31 ± 0.07
	1	0.94 ± 0.0	0.15 ± 0.01	0.95 ± 0.01	0.33 ± 0.08
	10	0.95 ± 0.0	0.16 ± 0.01	0.95 ± 0.01	0.37 ± 0.09
Poly	0.1	0.99 ± 0.0	0.47 ± 0.03	**0.98 ± 0.0**	0.66 ± 0.08
	1	**0.99 ± 0.0**	0.64 ± 0.04	**0.98 ± 0.0**	0.72 ± 0.08
	10	**0.99 ± 0.0**	**0.72 ± 0.06**	0.97 ± 0.01	**0.75 ± 0.07**
RBF	0.1	0.98 ± 0.0	0.27 ± 0.01	**0.98 ± 0.0**	0.5 ± 0.09
	1	**0.99 ± 0.0**	0.48 ± 0.02	**0.98 ± 0.0**	0.61 ± 0.09
	10	**0.99 ± 0.0**	0.62 ± 0.06	0.98 ± 0.01	0.67 ± 0.09

Table 7. RF method performance evaluation on 1:3 positive to negative sampling ratio of the CombTVir dataset.

Criterion	gini		log loss	
Max No, Features	log n	sqrt(n)	log n	sqrt(n)
Acc	**0.99 ± 0.0**	**0.99 ± 0.0**	**0.99 ± 0.0**	**0.99 ± 0.0**
MCC	**0.98 ± 0.01**	**0.98 ± 0.01**	0.98 ± 0.02	**0.98 ± 0.01**
AUC-ROC	**0.99 ± 0.0**	**0.99 ± 0.0**	**0.99 ± 0.0**	**0.99 ± 0.0**
AUPR	**0.99 ± 0.0**	**0.99 ± 0.0**	**0.99 ± 0.0**	**0.99 ± 0.0**

Table 8. RF method performance evaluation on 1:5 positive to negative sampling ratio of the CombTVir dataset.

Criterion	gini		log loss	
Max No, Features	log n	sqrt(n)	log n	sqrt(n)
Acc	**0.99 ± 0.0**	**0.99 ± 0.0**	**0.99 ± 0.0**	**0.99 ± 0.0**
MCC	0.97 ± 0.03	**0.97 ± 0.02**	**0.97 ± 0.02**	**0.97 ± 0.02**
AUC-ROC	0.99 ± 0.0	0.99 ± 0.0	0.99 ± 0.0	0.99 ± 0.0
AUPR	**0.99 ± 0.0**	**0.99 ± 0.0**	**0.99 ± 0.0**	0.99 ± 0.01

Table 9. Performance evaluation of the different configurations of the RF model on a 1:10 sample of the dataset

Criterion	Gini		log loss	
Max No, Features	log n	sqrt(n)	log n	sqrt(n)
Acc	**0.99 ± 0.0**	**0.99 ± 0.0**	**0.99 ± 0.0**	**0.99 ± 0.0**
MCC	**0.97 ± 0.01**	0.97 ± 0.03	0.97 ± 0.02	0.97 ± 0.02
AUC-ROC	**0.99 ± 0.0**	**0.99 ± 0.0**	**0.99 ± 0.0**	0.99 ± 0.01
AUPR	0.98 ± 0.01	0.99 ± 0.01	**0.99 ± 0.0**	0.98 ± 0.01

Table 10. Performance evaluation of the different configurations of the RF model on a 1:100 sample of the dataset.

Criterion	Gini		log loss	
Max No, Features	log n	sqrt(n)	log n	sqrt(n)
Acc	**0.99 ± 0.0**	**0.99 ± 0.0**	**0.99 ± 0.0**	**0.99 ± 0.0**
MCC	**0.92 ± 0.02**	**0.92 ± 0.02**	**0.92 ± 0.02**	**0.92 ± 0.02**
AUC-ROC	0.97 ± 0.02	**0.97 ± 0.01**	0.96 ± 0.02	0.97 ± 0.02
AUPR	0.93 ± 0.04	**0.93 ± 0.03**	**0.92 ± 0.03**	**0.93 ± 0.03**

Table 11. Performance evaluation of the different configurations of the RF model on a 1:500 sample of the dataset

Criterion	Gini		log loss	
Max No, Features	log n	sqrt(n)	log n	sqrt(n)
Acc	**0.99 ± 0.0**	**0.99 ± 0.0**	**0.99 ± 0.0**	**0.99 ± 0.0**
MCC	0.79 ± 0.02	**0.8 ± 0.04**	0.78 ± 0.05	0.79 ± 0.04
AUC-ROC	0.91 ± 0.02	0.9 ± 0.02	**0.92 ± 0.02**	**0.92 ± 0.02**
AUPR	0.76 ± 0.06	0.76 ± 0.05	**0.78 ± 0.05**	**0.78 ± 0.06**

3 Complete results of deep learning method (DRaW) on the CombTVir dataset

This section provides the Full results of the deep learning method(DRaW) on the proposed dataset in Table 12.

Table 12. Validation of the deep learning method(DRaW) on the CombTVir dataset

ratio	ACC	MCC	AUC-ROC	AUPR
1:3	0.94 ± 0.04	0.83 ± 0.16	0.97 ± 0.02	**0.95 ± 0.03**
1:5	0.96 ± 0.03	0.84 ± 0.21	0.96 ± 0.06	0.94 ± 0.06
1:10	0.98 ± 0.02	0.84 ± 0.25	0.97 ± 0.02	0.93 ± 0.04
1:100	**0.99 ± 0.0**	**0.87 ± 0.02**	**0.98 ± 0.01**	0.86 ± 0.04
1:500	**0.99 ± 0.0**	0.77 ± 0.04	0.92 ± 0.04	0.69 ± 0.06

4 Complete list of predicted drugs

Table 13. Predicted combinations of virus and antiviral by SVM and RF.

Antiviral1	Antiviral2	Virus	Frequency
acyclovir	brivudine	CMV	18
acyclovir	cidofovir	HSV-2	12
acyclovir	brincidofovir	CMV	11
acyclovir	cidofovir	HSV-1	9
acyclovir	zidovudine	HSV-1	6
acyclovir	zidovudine	HSV-2	6
adefovir	zidovudine	HBV	5
acyclovir	telbivudine	CMV	4
acyclovir	telbivudine	HSV-2	4
adefovir	brivudine	HBV	4
acyclovir	telbivudine	HSV-1	3
acyclovir	didanosine	CMV	3
acyclovir	foscarnet	VZV	3
acyclovir	adefovir	CMV	3
acyclovir	trifluridine	HSV-1	2
alisporivir	zidovudine	HBV	2
acyclovir	brincidofovir	VZV	2
acyclovir	brivudine	VZV	2
acyclovir	adefovir	HSV-2	2
acyclovir	adefovir	HSV-1	2
alisporivir	ribovirin	MERS-CoV	2
alisporivir	ribovirin	HCV	1
acyclovir	ribovirin	HSV-1	1
acyclovir	quercetin	CMV	1
acyclovir	maribavir	CMV	1
acyclovir	ribavirin	HSV-2	1
artemether	brivudine	HBV	1
artemether	brincidofovir	HSV-1	1
artemether	brivudine	HSV-2	1
artemether	brivudine	HSV-1	1
artemether	brincidofovir	CMV	1
artemether	brincidofovir	HBV	1
artemether	brincidofovir	FLUAV	1
artemether	brincidofovir	HCV	1
artemether	brivudine	HCV	1
artemether	brivudine	CMV	1
acyclovir	zidovudine	HBV	1
adefovir	vidarabine	HBV	1
adefovir	5-trifluorothymidine(tft)	HBV	1
adefovir	edoxudine	HBV	1
acyclovir	idoxuridine	CMV	1
acyclovir	gemcitabine	CMV	1
amantadine	ribovirin	FLUAV	1
adefovir	mmudr	HBV	1

Table 14. Predicted combinations of virus and antiviral by DRaW

Antiviral1	Antiviral2	Virus	Frequency
acyclovir	baloxavir	HBV	2
acyclovir	baloxavir	SARS-CoV	2
acyclovir	baloxavir	EV71	1
acyclovir	baloxavir	FLUAV	1
acyclovir	baloxavir	HEV	1
acyclovir	cenicriviroc	EBV	1
acyclovir	baloxavir	JUNV	1
acyclovir	atazanavir	CMV	1
acyclovir	baloxavir	YFV	1
acyclovir	baloxavir	HPV	1
acyclovir	baloxavir	HSV-2	1
acyclovir	baloxavir	DENV	1
acyclovir	baloxavir	KSHV	1
acyclovir	baloxavir	LASV	1
acyclovir	baloxavir	HIV-1	1
acyclovir	baloxavir	SFTSV	1
acyclovir	baloxavir	MERS-CoV	1
acyclovir	baloxavir	VZV	1
acyclovir	baloxavir	B19V	1
acyclovir	baloxavir	EBV	1
acyclovir	baloxavir	JCV	1
acyclovir	baloxavir	RVFV	1
acyclovir	cenicriviroc	HIV-1	1
acyclovir	baloxavir	FLUBV	1
acyclovir	baloxavir	CVB3	1
acyclovir	baloxavir	EBOV	1
acyclovir	baloxavir	VACV	1
acyclovir	baloxavir	HMPV	1
acyclovir	baloxavir	hPIV-3	1
acyclovir	docosanol	HIV-1	1
acyclovir	baloxavir	CMV	1
acyclovir	docosanol	ZIKV	1
acyclovir	ribovirin	ZIKV	1
acyclovir	quercetin	CMV	1
acyclovir	eucalyptol	CMV	1
acyclovir	tipranavir	CMV	1
acyclovir	foscarnet	EBOV	1
acyclovir	docosanol	EBV	1
acyclovir	foscarnet	EBV	1
amantadine	ribovirin	FLUAV	1
acyclovir	hydroxyurea	HSV-2	1
acyclovir	zidovudine	HSV-1	1
